# *Trichoderma reesei* Dehydrogenase, a Pyrroloquinoline Quinone-Dependent Member of Auxiliary Activity Family 12 of the Carbohydrate-Active Enzymes Database: Functional and Structural Characterization

**DOI:** 10.1128/AEM.00964-19

**Published:** 2019-11-27

**Authors:** Annick Turbe-Doan, Eric Record, Vincent Lombard, Rajender Kumar, Anthony Levasseur, Bernard Henrissat, Marie-Line Garron

**Affiliations:** a INRA, UMR1163, Biodiversité et Biotechnologie Fongiques, Aix-Marseille Université, Marseille, France; b Architecture et Fonction des Macromolécules Biologique, UMR 7257 CNRS, USC 1408, Aix Marseille Université, Marseille, France; c Department of Biological Sciences, King Abdulaziz University, Jeddah, Saudi Arabia; University of Illinois at Urbana—Champaign

**Keywords:** X-ray crystallography, carbohydrate, carbohydrate-active enzymes, fungi, glycobiology

## Abstract

Pyrroloquinoline quinone (PQQ) is an *ortho*-quinone cofactor of several prokaryotic oxidases. Widely available in the diet and necessary for the correct growth of mice, PQQ has been suspected to be a vitamin for eukaryotes. However, no PQQ-dependent eukaryotic enzyme had been identified to use the PQQ until 2014, when a basidiomycete enzyme catalyzing saccharide dehydrogenation using PQQ as a cofactor was characterized and served to define auxiliary activity family 12 (AA12). Here we report the biochemical characterization of the AA12 enzyme encoded by the genome of the ascomycete Trichoderma reesei (*Tr*AA12). Surprisingly, only weak activity against uncommon carbohydrates like l-fucose or d-arabinose was measured. The three-dimensional structure of *Tr*AA12 reveals important similarities with bacterial soluble glucose dehydrogenases (sGDH). The enzymatic characterization and the structure solved in the presence of calcium confirm the importance of this ion in catalysis, as observed for sGDH. The structural characterization of *Tr*AA12 was completed by modeling PQQ and l-fucose in the enzyme active site. Based on these results, the AA12 family of enzymes is likely to have a catalytic mechanism close to that of bacterial sGDH.

**IMPORTANCE** Pyrroloquinoline quinone (PQQ) is an important cofactor synthesized by prokaryotes and involved in enzymatic alcohol and sugar oxidation. In eukaryotes, the benefit of PQQ as a vitamin has been suggested but never proved. Recently, the first eukaryotic enzyme using PQQ was characterized in the basidiomycete Coprinopsis cinerea, demonstrating that fungi are able to use PQQ as an enzyme cofactor. This discovery led to the classification of the fungal PQQ-dependent enzymes in auxiliary activity family 12 (AA12) of the Carbohydrate-Active Enzymes (CAZy) database (www.cazy.org) classification. In the present paper, we report on the characterization of the ascomycete AA12 enzyme from Trichoderma reesei (*Tr*AA12). Our enzymatic and phylogenetic results show divergence with the only other member of the family characterized, that from the basidiomycete Coprinopsis cinerea. The crystallographic structure of *Tr*AA12 shows similarities to the global active-site architecture of bacterial glucose dehydrogenases, suggesting a common evolution between the two families.

## Abstract (precis)

Pyrroloquinoline quinone (PQQ) is an important cofactor synthesized by prokaryotes and involved in enzymatic alcohol and sugar oxidation. In eukaryotes, the benefit of PQQ as a vitamin has been suggested but never proved. Recently, the first eukaryotic enzyme using PQQ was characterized in the basidiomycete Coprinopsis cinerea, demonstrating that fungi are able to use PQQ as an enzyme cofactor. This discovery led to the classification of the fungal PQQ-dependent enzymes in auxiliary activity family 12 (AA12) of the Carbohydrate-Active Enzymes (CAZy) database (www.cazy.org) classification. In the present paper, we report on the characterization of the ascomycete AA12 enzyme from Trichoderma reesei (*Tr*AA12). Our enzymatic and phylogenetic results show divergence with the only other member of the family characterized, that from the basidiomycete Coprinopsis cinerea. The crystallographic structure of *Tr*AA12 shows similarities to the global active-site architecture of bacterial glucose dehydrogenases, suggesting a common evolution between the two families.

## INTRODUCTION

Pyrroloquinoline quinone (PQQ) is an *ortho*-quinone cofactor that was discovered at the end of the 1970s in a bacterial methanol dehydrogenase (1). Until recently, enzymes using PQQ as a cofactor were thought to be found only in prokaryotes. Intriguingly, however, the distribution of PQQ is not limited to prokaryotes, as it was also found in various plants and animals (2–4). The function of PQQ in eukaryotes is not clear, but experiments have shown that PQQ is necessary in the diet for the correct development of mice (5). The hypothesis that eukaryotes could use PQQ as a vitamin has been vigorously debated, but eukaryotic PQQ-dependent enzymes remained elusive (6, 7).

In bacteria, the PQQ-dependent dehydrogenases are mainly synthesized by Gram-negative bacterial strains. These enzymes are involved in alcohol dehydrogenation, such as by methanol dehydrogenases (MDHs), or sugar dehydrogenation, such as by glucose dehydrogenases (GDHs) (8). GDHs can be divided into two distinct families, namely, soluble GDHs (sGDHs) and membrane-bound GDHs (mGDHs), with the latter being closely related to the MDH family (8). No three-dimensional structure of mGDH has been determined yet, but the sequence similarity with MDHs has allowed the modeling of an eight-blade β-propeller domain and the identification of the putative catalytic residues (9). The second family, sGDH, corresponds to soluble GDHs, which fold as a six-blade β-propeller (10). The active site of sGDH is wide, commensurate with the broad substrate specificity of the enzyme (11), and it was shown that sGDHs use a general base-catalyzed hydride transfer assisted by calcium (12).

Carbohydrate-active enzymes (CAZymes) are classified in sequence-based families in the Carbohydrate-Active Enzymes (CAZy) database (www.cazy.org) (13). A new category of CAZymes, named auxiliary activity (AA) enzymes, was recently introduced into the CAZy database after the oxidative cleavage of glycosides was established (13–16). In 2014, auxiliary activity family 12 (AA12) was created after the characterization of a pyranose dehydrogenase (PDH) from the basidiomycete Coprinopsis cinerea (*Cc*PDH) (17). Interestingly, *Cc*PDH used PQQ as a cofactor to oxidize unusual monosaccharides, such as d-glucosone or l-fucose (17, 18). The discovery and characterization of *Cc*PDH established that some eukaryotes and, in particular, fungi possess enzymes that use PQQ as a cofactor. To better understand the function of the AA12 family of enzymes in fungi, we performed a phylogenetic analysis of the family along with a functional and structural study of AA12 from the ascomycete Trichoderma reesei (*Tr*AA12), a fungus heavily utilized as the industrial model for cellulose degradation (19). The structure of *Tr*AA12 reveals interesting structural similarities with bacterial sGDHs. We combined the crystallographic structures and molecular docking simulations to explain the binding of PQQ and of the substrate in the catalytic binding site. The conservation of the catalytic machinery indicates that *Tr*AA12 probably uses the same general base-catalyzed hydride transfer observed for sGDH.

## RESULTS

### Sequence analysis of family AA12.

**FIG 1:**
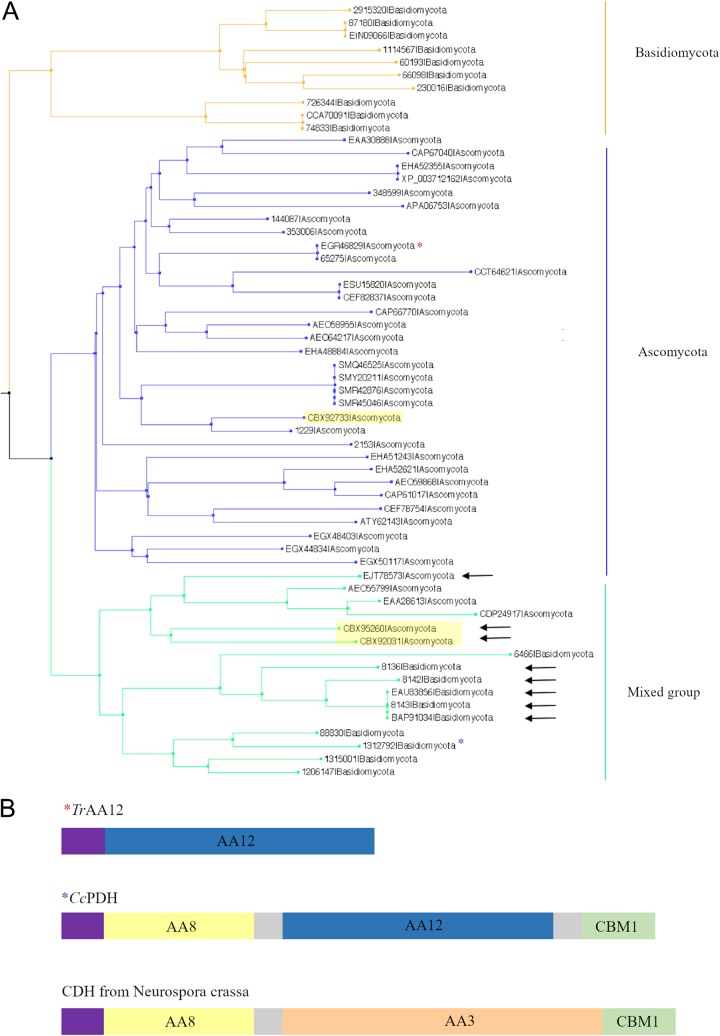
Phylogenetic tree of the AA12 family. (A) The phylogenetic tree is subdivided into three subgroups, colored orange for the Basidiomycota group, blue for the Ascomycota group, and green for the mixed group. The proteins are identified by their GenBank accession numbers or their JGI protein identifiers. The red and blue asterisks correspond to the *Tr*AA12 and the *Cc*PDH sequences, respectively. The black arrows indicate the multimodular sequences having at least both the AA12 and the AA8 domains. The three sequences from Leptosphaeria maculans are highlighted in yellow. (B) Schematic of the modularity of *Tr*AA12, *Cc*PDH, and CDH from Neurospora crassa (UniProt accession number Q7S0Y1). Signal peptides are in purple, linkers are in gray, AA12 domains are in dark blue, AA8 is in yellow, AA3 is in orange, and CBM1 is in green.

The phylogenetic analysis of family AA12 performed on 59 fungal sequences (20, 21) subdivided the family into three subgroups (Fig. 1A). Two groups directly reflect the taxonomy; i.e., they coincide exclusively with the Ascomycota and the Basidiomycota, respectively. The third subgroup contains sequences from both phyla. Interestingly, several copies of family AA12 sequences which are encoded by the same genome can be found in separate subgroups of the phylogenetic tree. For example, the genome of the ascomycete Leptosphaeria maculans codes for three AA12 enzymes; one is in the Ascomycota subgroup, whereas the two others are in the mixed subgroup. The two sequences of L. maculans that are in the mixed subgroup present a bimodular organization with an AA8 domain fused to the N terminus of the AA12 domain (Fig. 1A). Sequence analysis also shows that the AA8 domains and family 1 carbohydrate-binding modules (CBM1) are the only domains that are appended, together or not, to the AA12 domain. When present, the AA8 domain is always found at the N terminus of the AA12 domain, while CBM1 is appended to the C terminus (Fig. 1B). The AA8 domain is a fungal *b*-type cytochrome which uses a heme as a cofactor (22). Family AA8 was initially characterized as a domain in cellobiose dehydrogenases (CDHs), where it is appended to an AA3 subfamily 1 (AA3_1) domain (23). In CDHs, AA8 is implicated in electron transfer during the dehydrogenation of the substrate by AA3_1 (24). Interestingly, AA3_1 proteins present the same modularity observed for AA12, i.e., AA8 at the N terminus and a CBM1 at the C terminus of the AA3_1 domain (Fig. 1B). The CBM1 module is a small peptide, mainly found in fungi, which was initially identified to be a cellulose-binding domain (25). This domain can be appended to polypeptides in various families of glycoside hydrolases (GHs) and polysaccharide lyases (PLs), as well as AA domains. The bimodular architectures containing AA8 and AA12 are strictly found in the mixed subgroup of the phylogenetic tree (Fig. 1A). The presence of a signal peptide in most AA12 sequences suggests that these enzymes are secreted. Several AA12 sequences harbor a C-terminal transmembrane domain or a glycosylphosphatidylinositol anchor, presumably attaching the protein to the cell outer surface. The AA12 protein of T. reesei belongs to the Ascomycota clade (Fig. 1A). Unlike *Tr*AA12, *Cc*PDH is a multimodular protein composed of three domains, where the AA12 domain is flanked by an AA8 domain and a CBM1 domain (Fig. 1B) (17). Among the analyzed sequences, this modularity with three domains is rarely observed and seems to occur mainly in the Agaricales order of the Basidiomycota. On the other hand, the bimodular structure containing the AA8 domain and the AA12 domain cannot be linked to a particular taxonomic clade and is observed more frequently than the AA8-AA12-CBM1 organization (Fig. 1A).

### Biochemical characterization of *Tr*AA12.

After the purification of *Tr*AA12, sodium dodecyl sulfate-polyacrylamide gel electrophoresis (SDS-PAGE) revealed a single band with a molecular mass of approximately 75 kDa (data not shown), much higher than the theoretical molecular mass of 45.3 kDa, based on the amino acid sequence from residues 24 to 435. Seven N-glycosylation sites were predicted based on the consensus sequence [Asn-X-(Ser/Thr)] on Asn25, Asn94, Asn147, Asn184, Asn228, Asn306, and Asn341. The glycosylation was confirmed by SDS-PAGE following peptide-*N*-glycosidase treatment (data not shown) and was observed on Asn94, Asn147, Asn184, Asn306, and Asn341 in the crystallographic structures.

**FIG 2:**
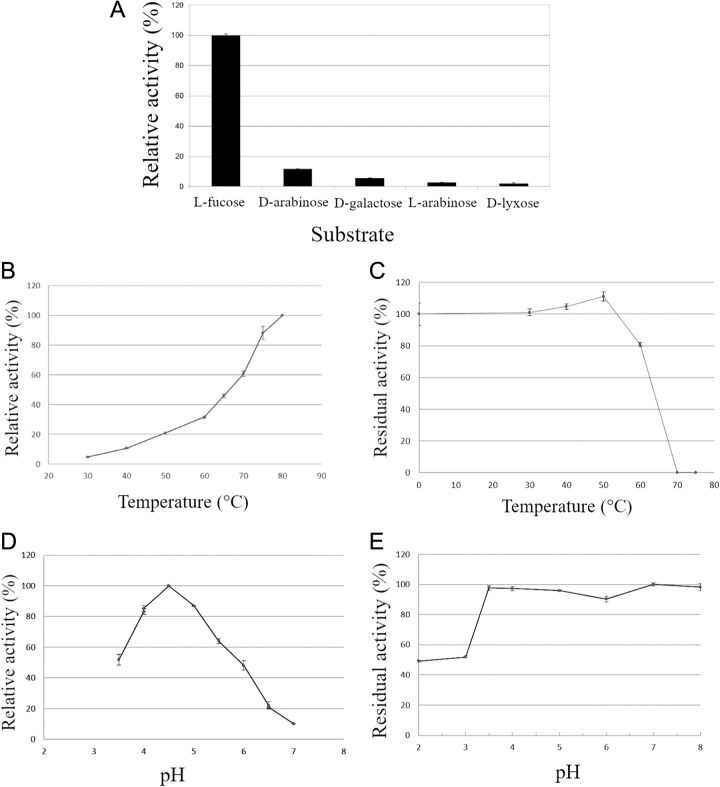
Enzymatic characterization of *Tr*AA12. (A) Specificity of *Tr*AA12 against various substrates in the presence of 500 mM the electron donor. The activities are given relative to the activity of l-fucose. (B to E) Effects of temperature and pH on the activity of the purified *Tr*AA12. Various temperatures ranging from 30°C to 80°C and various pH values (3.5 to 7) were tested under standard conditions. The effects of temperature (C) and pH (E) on the stability of the purified protein after 1 h in 100 mM tartrate buffer (pH 2 to 3), 100 mM sodium acetate buffer (pH 3.5 to 6), 100 mM HEPES (pH 7 to 8) are shown. All assays were performed with l-fucose as the substrate.

Various monosaccharides were tested to evaluate the enzyme activity and specificity. Several monosaccharides were tested under standard conditions requiring calcium and PQQ as cofactors. No detectable activity against d-glucose or d-fucose was found. *Tr*AA12 preferentially oxidizes l-fucose, and weak activity was measured against d-arabinose (11.6% relative activity), d-galactose (5.7%), l-arabinose (2.8%), and d-lyxose (2.2%) (Fig. 2A). The activity against d-glucosone was also tested, but the activity of *Tr*AA12 was 10 times lower than that measured with l-fucose. Unfortunately, the reductive activity of d-glucosone on cytochrome *c* was not stable enough to yield reliable activity. The kinetic constants determined at steady state for l-fucose under standard conditions revealed a *K_m_* of 0.0999 ± 0.0097 M and a *k*_cat_ of 0.012 ± 0.0003 s^−1^, resulting in a low catalytic efficiency (*k*_cat_/*K_m_*) of 0.119 s^−1^ M^−1^. These kinetic data assume the full occupancy of the cofactor, which, under these experimental conditions, remains uncertain. The temperature profile was determined up to 80°C, and *Tr*AA12 activity increased gradually with temperature between 30 and 80°C (Fig. 2B). The thermostability of the purified *Tr*AA12 was tested over a range of temperatures of from 0 to 75°C. *Tr*AA12 was stable up to 50°C, and its activity decreased by 20% after heating at 60°C for 30 min. No activity was found after 30 min of incubation at 70°C (Fig. 2C). The optimal pH was 4.5. When the pH was below 3.5 and above 7, only 50% and 10% of the maximum activity, respectively, was attained (Fig. 2D). For stability in relation to pH, *Tr*AA12 was stable from pH 3.5 to pH 8. Below pH 3.5, only 50% of the activity could be measured (Fig. 2E).

### Three-dimensional structures of *Tr*AA12.

**FIG 3:**
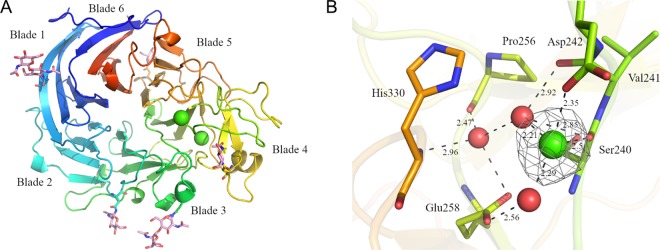
Crystallographic structure of *Tr*AA12. (A) The structure of *Tr*AA12 presented is the one solved with the two calcium ions (PDB accession number 6I1T), which are represented by green spheres. The structure is colored from blue (N terminus) to red (C terminus). The glycosylation observed in the structure is represented as pink sticks. (B) The coordination network stabilizing the putative catalytic calcium. The amino acids are in stick representation, and the red spheres represent the water molecules. The black dotted lines represent electrostatic forces, and distances were measured by use of the program Coot (50). The electron density observable on the 2*F*_o_ − *F*_c_ map (where *F*_o_ and *F*_c_ are the observed and the calculated structure factors, respectively) after refinement is represented in a mesh contoured at 1.5σ.

Three structures of *Tr*AA12 were solved, namely, the structure of the iodide adduct for phasing, that of the native protein, and that of the catalytic calcium-containing protein (PDB accession numbers 6I1Q, 6H7T, and 6I1T, respectively). The three structures superimposed very well, and no major differences in the main chains were observed. The polypeptide chain observable in the crystal structure of *Tr*AA12 covers amino acids Gln24 to Asn435. This domain folds as a 6-blade β-propeller (Fig. 3A). This fold is frequently found in various enzyme families, where the number of blades varies from 4 to 12 (26). According to the common terminology, each blade of *Tr*AA12 is numbered from 1 to 6 and contains four antiparallel β-strands, named β-strands A to D (Fig. 3A). All the structures solved present a constitutive calcium. This calcium stabilizes a long loop which joins sheets C and D of the fourth blade. The calcium ion presents a typical octahedral coordination. Three amino acids are directly involved in the coordination: Glu259 and Gln301 with their side chains and Tyr276 by the main chain. Three water molecules complete the coordination, stabilized mainly by the side chains of Glu238 and Asn26. Of the seven predicted N-glycosylation sites, five are visible in the density maps on Asn94, Asn147, Asn184, Asn306, and Asn341. The density map quality varies between the structures, and the last sugars are built on partial density. The two longer N-glycans, on Asn94 and Asn306, come close to the symmetric molecule and participate in crystal packing.

**FIG 4:**
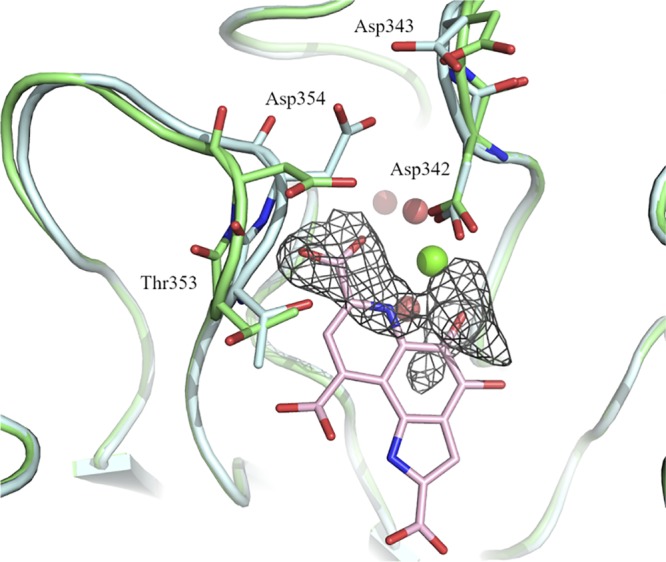
Superimposition of the native and the calcium-bound structures. The native structure is in green (PDB accession number 6H7T), and the structure with catalytic calcium is in cyan (PDB accession number 6I1T). The docked PQQ is shown in a stick representation and in pink. The residual electron density observable on the *F*_o_ − *F*_c_ map after refinement is represented in a mesh contoured at 3σ.

To understand the enzymatic mechanism, we tried to solve the structure of *Tr*AA12 in the presence of the cofactor and of the substrate. Soaking experiments were performed on native crystals in crystallization buffer supplemented with calcium, PQQ, and l-fucose. Several crystal soaking experiments were performed, and eight data sets were collected. A large additional electron density inside a pocket and a reorganization of the amino acids surrounding this electron density were observed in each of the eight structures (Fig. 4). Only the best-resolution structure was deposited in the Protein Data Bank (PDB) and is presented here (PDB accession number 6I1T). Based on the difference map peak intensity, a part of the additional electron density was assigned to a second calcium ion. The plausibility of calcium was confirmed by the web server CheckMyMetal (27). Without the cofactor, the coordination sphere is uncompleted but is predicted to be octahedral. The root mean square deviation (RMSD) observed in the angle geometry (gRMSD) was 13.9°, which is only 0.4° higher than the acceptable value for this parameter. The coordination distances measured and compared to the distances of the structures in the Cambridge Structural Database (CSD) are in agreement with those for a calcium ion (Fig. 3B). Finally, after refinement, the calcium presents a B-factor of 33.9 Å^2^, which is coherent with the average of 38.4 Å^2^ for its environment. The calcium is directly coordinated by the side chain of Asp242 and the carbonyl of the Ser240 and is completed by two water molecules. A third water molecule completes this well-organized network (Fig. 3B). This organization is conserved in the native crystal, where the calcium ion is replaced by a water molecule.

The addition of the catalytic calcium and its coordinating water molecules was not sufficient to fully fit the additional density. The remainder of the density was too large and too dense to be filled only by water molecules (Fig. 4) but too small to correspond to the entire PQQ molecule, presumably indicating a weak and partial occupancy of the site (Fig. 4). In addition to PQQ, several molecules were tested to occupy the extra electron density, but no satisfactory result was obtained. As the occupancy was too weak to place the PQQ molecule without ambiguity, the PDB file was deposited only with the catalytic calcium and without PQQ (PDB accession number 6I1T). Even if the additional density cannot be clearly assigned to PQQ, the molecule that enters the active site induced the movement and the reorganization of the neighboring amino acids (Fig. 4). The superimposition of the catalytic calcium structure with the native one shows a movement of the loop spanning residues Thr353 to Thr357. This shift involves the reorientation of several side chains and the creation of new hydrogen bonds (Fig. 4). Asp354 presents the largest difference, with a displacement of 1.55 Å of its C-α and a change of the orientation of its side chain. The new side chain position of Asp354 is stabilized by two new hydrogen bonds with Asp243 (Fig. 4). These movements partially close a part of the binding site and bring the side chains of Asp242 and Thr353 near the putative substrate binding site.

### Structural homologs.

**FIG 5:**
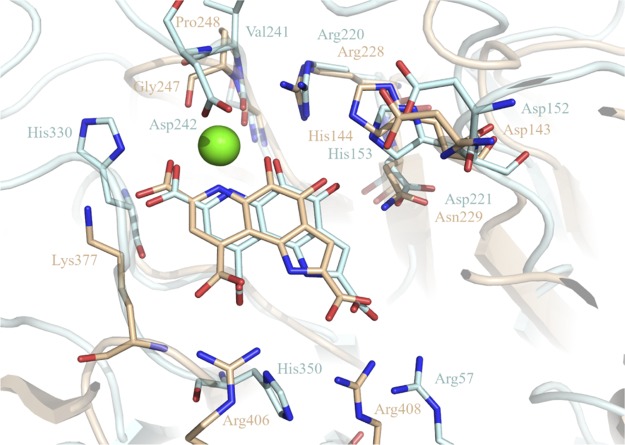
Structural similarity. Superimposition of the *Tr*AA12 docking structure with that of sGDH from Acinetobacter calcoaceticus in the presence of PQQ (PDB accession number 1CQ1). The calcium ions of the two structures are perfectly superimposed and represented as green spheres, amino acids and PQQ are in stick representation, *Tr*AA12 is in cyan, and sGDH is in orange.

Structural homologs of *Tr*AA12 were searched with the Dali server (28). The closest structures (with Z-scores of about 30) were from the bacterial soluble glucose dehydrogenase (sGDH) family. sGDHs use PQQ as their cofactor and utilize a calcium-assisted mechanism to oxidize mono- or disaccharides to the corresponding lactones (29). Several structures of sGDHs are available in PDB, but only the structure of the Acinetobacter calcoaceticus enzyme (PDB accession number 1CQ1) with the catalytic calcium, the PQQ, and the substrate was solved (12). The superimposition of *Tr*AA12 with A. calcoaceticus sGDH shows that the global fold is well conserved (RMSD, 2.4 Å with an alignment done on 284 amino acids with 17% sequence identity). Three calcium sites were identified in the A. calcoaceticus sGDH structure, but only two sites are strictly conserved in the other sGDH structures (30, 31). Interestingly, the two sites are the same as those observed in the *Tr*AA12 structure. The first calcium is the constitutive calcium, which has a well-conserved coordination. Except for Gln301 in *Tr*AA12, which is replaced by a water molecule in sGDH, all the others amino acids involved in the calcium coordination, directly or indirectly via water molecules, are strictly conserved. The second calcium is involved in catalysis and interacts with PQQ in the binding site of sGDHs. The coordination of the second calcium of *Tr*AA12 is comparable to that observed in A. calcoaceticus sGDH (Fig. 5). In the two structures, the loop joining sheet B to sheet C of the fourth blade is important for the coordination of the calcium (Fig. 3A). In the A. calcoaceticus sGDH structure, Gly247 and Pro248 in this loop stabilize the calcium ion. In *Tr*AA12, Ser240 adopts the same role and position as Gly247 in A. calcoaceticus sGDH. Because *Tr*AA12 has a longer loop than A. calcoaceticus sGDH, the amino acid which directly follows Ser240 is not well positioned to interact with the calcium like the Pro248 in sGDH. Instead, the long loop of *Tr*AA12 allows the side chain of Asp242 to complete the calcium coordination and replace one of the water molecules observed in the calcium coordination of A. calcoaceticus sGDH (Fig. 5).

### Molecular docking of PQQ and l-fucose on *Tr*AA12.

**FIG 6:**
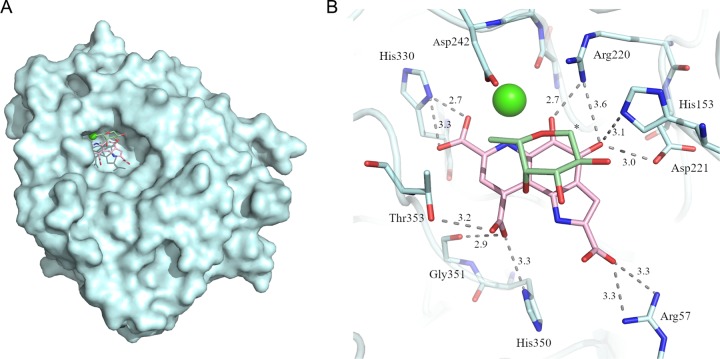
Molecular docking of PQQ and l-fucose. (A) The overall structure of *Tr*AA12 in the presence of the catalytic calcium is represented as a cyan surface. The molecules docked in the putative active site are indicated in stick representation and are in pink for PQQ and in green for l-fucose. The calcium ion is observable at the bottom of the cavity, whereas PQQ is largely exposed to the solvent. (B) The amino acids potentially involved in PQQ and the substrate interaction are shown in stick representation. The black dashed lines represent electrostatic forces, and distances were measured by use of the program Coot (50). An asterisk indicates carbon 1 of l-fucose.

Because no density was observed for l-fucose and only a partial density was obtained for PQQ in our crystals, molecular docking studies were initiated to confirm the binding position of PQQ and to estimate the putative position of the substrate. The docking simulation was done in two steps. First, PQQ was docked and the best protein-cofactor complex was kept. Then, the best substrate position was calculated using the protein-cofactor complex. The best-docked position of PQQ is close to the catalytic calcium. The PQQ molecule occupies a large flat cavity in front of the calcium pocket (Fig. 6A). The oxygen (O-7A) of the carboxylate, the nitrogen (N-6) of the amine, and the oxygen (O-5) of the ketone are positioned 2.9, 3, and 2.5 Å, respectively, from the calcium. These distances are compatible with electrostatic interactions which could be involved in calcium stabilization and activation. The PQQ cofactor is surrounded mainly by basic amino acids. The side chains of Arg57, His153, Arg220, His330, and His350 are well positioned (with distances of less than or close to 3 Å) for electrostatic and/or hydrogen bonds with the carboxylates or the carbonyl groups of PQQ. Hydrogen bonds with two acidic residues (Asp221 and Asp242) and the amino groups of the main chain of Thr353 and Asp354 complete the interaction (Fig. 6B). The floor of the PQQ cavity is constituted by the side chains of Asn239 and Ser332. On the other side, the PQQ molecule is totally exposed to the solvent and no amino acid hinders substrate accessibility (Fig. 6A). In conclusion, the docking simulation suggests that the putative substrate binding site is located above the PQQ molecule in the same cavity. The l-fucose residue docks onto the PQQ surface by hydrophobic stacking at a distance of about 4 Å. The sugar molecule could be stabilized by a hydrogen bond between the hydroxyl group at C-2 and the side chain of Asp152. In l-fucose, the C-4 hydroxyl group is in an axial position and points down toward the PQQ cofactor. This orientation allows the establishment of a hydrogen bond with the carboxylic function at C-9 of PQQ. Finally, the methyl group of Thr353 comes close (3.5 Å) to the methyl group of l-fucose (Fig. 6B).

## DISCUSSION

Family AA12 was created in 2014 after the biochemical characterization of the Coprinopsis cinerea pyranose dehydrogenase (*Cc*PDH) (17). Whereas PQQ was considered a strictly prokaryotic cofactor, the discovery of family AA12 demonstrated that some eukaryotes, at least fungi, can use PQQ for monosaccharide dehydrogenation processes. Despite a poor sequence identity (<20%), *Tr*AA12 presents important structural similarities with bacterial sGDHs, including the functional and the structural calcium sites. The PQQ molecule could not be built in the *Tr*AA12 structure because only a partial density was observed. Docking simulations placed the PQQ molecule in a position consistent with the extra density observed in the crystal structure (Fig. 4). This extra density could correspond to the carboxylic group (C-7) and the nitrogen (N-6) of the pyridine cycle and may be completed by the oxygen of the ketone (C-5). This part of the cofactor is stabilized by the interaction with the calcium, providing a possible explanation for the residual density observable only around the calcium ion. The remainder of the PQQ molecule could be more mobile around this hinge. The predicted PQQ-binding site is comparable to that observed in A. calcoaceticus sGDH, where the cofactor is stabilized mainly by electrostatic bonds involving well-conserved basic amino acids (Fig. 5). As mentioned above, the structure solved in the presence of calcium and PQQ shows a small loop movement and a side chain reorientation. The loop going from Thr353 to Thr357 comes closer to the extra density that could correspond to the carboxylic group (C-7) of PQQ. In this position, the carboxylic group of PQQ can establish hydrogens bonds with the amines of the main chains of Thr353 and Asp354, which would explain the movement of the loop. This reorganization results in a narrower substrate binding site (Fig. 4).

**FIG 7:**
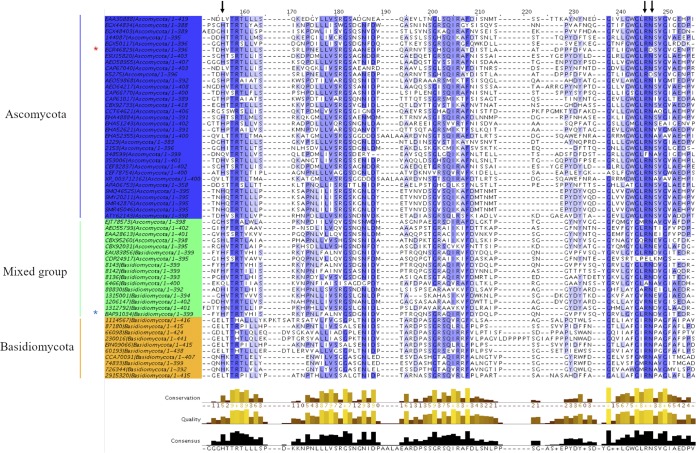
Sequence alignment. The red and blue asterisks correspond to the *Tr*AA12 and the *Cc*PDH sequences, respectively. The black arrows indicate the putative catalytic amino acids His153, Arg220, and Asn221 in *Tr*AA12.The amino acids are colored based on percent identity.

In the sGDH family, the oxidation mechanism involves a direct hydride transfer between substrate C-1 and the C-5 of the PQQ, followed by a general base-catalyzed proton abstraction (12). Based on the quaternary structure of A. calcoaceticus sGDH, His144 is the best candidate catalytic base, functionally assisted by calcium and Arg228 to enhance the reactivity of the C-5—O-5 bond of PQQ (12, 32). The orientation of the l-fucose, found by docking simulation on *Tr*AA12, is the same as that observed for the d-glucose in the structure of A. calcoaceticus sGDH (PDB accession number 1CQ1) (12). The proton at the C-1 position of the l-fucose is well oriented and points down toward C-5 of PQQ, which would be coherent with a catalytic mechanism similar to that of sGDH. Moreover, the equivalent of His144 in A. calcoaceticus sGDH, His153, is close enough to the anomeric carbon of l-fucose (3 Å) to act as the general base. In addition to the catalytic histidine, the two amino acids that surround the PQQ ketones (Arg228 and Asn229 in A. calcoaceticus sGDH) and that are potentially important for the catalytic activity are also conserved in *Tr*AA12 (Arg220 and Asp221). If *Tr*AA12 operates with the same catalytic mechanism as sGDH, then His153 would be the catalytic base. However, phylogenetic analysis showed that this histidine is not strictly conserved in family AA12 (Fig. 7). In more than 25% of the sequences, this histidine was not conserved and was frequently replaced by a leucine. Interestingly, the conservation rate of His153 was directly linked to the subgroup classification. More than 93% of the sequences of the mixed subgroup harbored the conserved putative catalytic histidine, whereas the rate dropped to 78% for the sequences belonging to the Ascomycota and to only 30% for the sequences belonging to the Basidiomycota. In comparison, the pair Arg/Asx (Arg220 and Asp221 in *Tr*AA12), which completes the PQQ interaction with the reactive ketones, was more conserved (about 94% for each) (Fig. 7). The conservation of the Arg/Asx dyad in family AA12 suggests that these amino acids are involved in catalysis. The structural similarities observed between *Tr*AA12 and sGDH suggest that the two families have a common evolutionary ancestor. However, the high sequence divergence around the putative catalytic residue in some subgroups raises questions about the conservation of the catalytic mechanism and/or of the substrate specificity/selectivity.

So far, family AA12 is the first and the only known eukaryotic family using PQQ as a cofactor. However, fungi do not possess the machinery to synthesize PQQ. This was also previously observed for some bacterial strains. For example, Escherichia coli is not able to synthesize PQQ but produces a PQQ-dependent dehydrogenase (33). The wide natural availability of PQQ allows these enzymes—which are extracellular—to find the cofactor directly in the medium (34). For E. coli, the PQQ dehydrogenase is involved in its periplasmic respiratory network. The capacity to use PQQ in an electron transfer system increases the bioenergetics options of the organisms in various environments (31). The metabolic pathway in which fungal family AA12 would be involved is unclear at present. Moreover, the substrates oxidized by family AA12 enzymes are uncommon and naturally infrequent. The oxidoreduction mechanism used by lytic polysaccharide monooxygenases (LPMOs) to degrade recalcitrant polysaccharides requires an electron transfer system (35). LPMOs can accept electrons from various donors. This versatility is utilized by fungi to break down the biomass at various steps of degradation (35). Among the electron donors of LPMOs, several families of auxiliary activity enzymes are suspected to play a role. The best characterized one is cellobiose dehydrogenase (CDH), which has a modular structure comprising an N-terminal family AA8 domain appended to a family AA3 domain, sometimes followed by a C-terminal CBM1 domain (36). Functional and structural data have shown electron transfer from domain AA3 to domain AA8 of CDH, followed by LPMO reduction to initiate oxygen activation (37). Interestingly, some AA12 enzymes, like *Cc*PDH, display a modular organization similar to that of CDH. Our phylogenetic analysis shows that the evolutionary pressure on modular AA12 proteins was different, since, regardless of the phylum, multimodular enzymes are gathered in a separate subgroup and present a higher rate of conservation of the putative catalytic histidine. A recent publication has confirmed that family AA12 enzymes associated with an AA8 domain can activate AA9 LPMOs (38). It is thus possible that a multimodular AA12, like *Cc*PDH, could represent one of the links of the extracellular electron transfer chain that regulates LPMO activity independently of changes in the substrate composition. However, this hypothesis cannot be generalized to the entire AA12 family, as the majority of AA12 sequences are nonmodular. Moreover, experimentally, because of its low enzymatic rate and different specificity, *Tr*AA12 diverges from *Cc*PDH. *Tr*AA12 also seems to have an affinity for PQQ weaker than that previously reported for *Cc*PDH (17).

In conclusion, the clades observed on the phylogenetic tree of family AA12 may reflect different functional activities within these subfamilies. Further investigations are needed to identify the physiological substrate of *Tr*AA12 compared to that of *Cc*PDH. Nevertheless, the important structural conservation observed between *Tr*AA12 and the PQQ enzymes on which bacteria depend provides support for our hypothesis that *Tr*AA12 requires PQQ as a cofactor.

## MATERIALS AND METHODS

### Strains and culture media.

Escherichia coli JM109 (Promega) was used for the construction and propagation of vectors, and Aspergillus niger strain D15#26 (lacking the *pyrG* gene) was used for the production of the recombinant protein (39). After cotransformation with vectors containing, respectively, the *pyrG* gene and the expression cassette containing the *Tr*AA12-encoding gene, A. niger was grown for selection on solid minimal medium (without uridine) containing 70 mM NaNO_3_, 7 mM KCl, 11 mM KH_2_HPO_4_, 2 mM MgSO_4_, 1% (wt/vol) glucose, and trace elements (a 500× stock containing 38 mM ZnSO_4_, 12.5 mM MnCl_2_, 9 mM FeSO_4_, 3.55 mM CoCl_2_, 3.2 mM CuSO_4_, 3.1 mM Na_2_MoO_4_, a 87 mM EDTA). In order to screen the transformants for the production of the recombinant protein, 100 ml of culture medium containing 70 mM NaNO_3_, 7 mM KCl, 200 mM Na_2_HPO_4_, 2 mM MgSO_4_, 5% (wt/vol) glucose, and trace elements was inoculated with 2 × 10^6^ spores ml^−1^ in a 500-ml baffled flask.

### Expression vector construction and fungal transformation.

The sequence encoding amino acids 24 to 435 of the T. reesei family AA12 protein (*Tr*AA12; GenBank accession number EGR46829.1) was codon optimized for A. niger, synthesized by GeneArt Gene Synthesis (Life Technologies, Germany), and cloned into pAN52.3 (GenBank accession number Z32689) by using restriction enzymes NcoI and HindIII. In the expression vector, the A. nidulans glyceraldehyde-3-phosphate dehydrogenase gene (*gpdA*) promoter, the 5′ untranslated region of *gpdA* mRNA, and the A. nidulans *trpC* terminator were used to drive the expression of the gene coding for *Tr*AA12.

Fungal cotransformations were carried out as described by van den Hondel and colleagues using the expression vectors and pAB4-1 containing the *pyrG* selection marker in a 5:1 ratio (40, 41). In addition, A. niger D15#26 was transformed with the *pyrG* gene without the expression vector for the control experiment. Cotransformants were selected for uridine prototrophy on selective minimal medium plates (without uridine) and incubated for 5 days at 30°C. In order to screen the transformants, two methods were used to screen 17 positive transformants. First, protoplasts were prepared from the transformant spores in caylase (20 mg ml^−1^; Cayla-InvivoGen) at 30°C for 3 h. Then, a PCR method was performed on the derived protoplasts by using specific primers (forward primer 5′-AACAGACCAACACCGTCGATCC-3′ and reverse primer 5′-CAACAAAGTTGGTGTTGCAGGTCGCG-3′). Second, positive transformants were cultured in liquid medium and aliquots (350 μl) were collected after 7 days. Cells were pelleted by centrifugation (5 min at 10,000 × *g*), the resulting supernatant was concentrated onto microcentrifugal units (Spin-X centrifuge tube filters; Corning) with a 10-kDa cutoff, and protein production was confirmed by 10% sodium dodecyl sulfate-polyacrylamide gel electrophoresis (SDS-PAGE).

### Phylogenetic analysis.

Phylogenetic analysis was performed on 59 fungal sequences downloaded from GenBank and from the fungal genome portal of the JGI (https://mycocosm.jgi.doe.gov/mycocosm/home). All selected sequences were from published genomes (20, 21). The amino acid sequences were aligned by use of the MAFFT program (42). We used the option –maxiterate 1000 and –global pair to obtain high accuracy, where –maxiterate [number] indicates the maximum number of iterative refinement and –global pair forces global pairwise alignment. To generate the tree, we built a matrix of maximum likelihood distances based on model-of-substitution LG (43). Finally, the phylogeny reconstruction tree was created using the BIONJ program (44).

### Purification of recombinant protein.

The best isolate corresponding to the transformant that produced an intense protein band was inoculated under the same conditions described above for the screening procedure. The culture was harvested after 8 days of growth, filtered (0.22-μm pore size), and concentrated by ultrafiltration through a polyethersulfone membrane (molecular mass cutoff, 10 kDa; Millipore). The concentrated fraction was dialyzed against a 50 mM Tris-HCl (pH 7.0) binding buffer, and the purification of the His-tagged proteins was performed on a HisTrap HP column (GE Healthcare Life Sciences) (45).

### Enzymatic assays and kinetics properties.

Dehydrogenase activity was determined at 40°C in a dye-linked system containing cytochrome *c* by spectrophotometry at 550 nm (ε_550_ = 28 mM^−1 ^cm^−1^). The standard reaction condition consisted of cytochrome *c* (50 μM), sodium acetate buffer (50 mM, pH 4.5), l-fucose (500 mM), PQQ (4 μM), and CaCl_2_ (1 mM) with 4.6 μM purified enzymes.

The dehydrogenase activity of the purified *Tr*AA12 protein was assayed at various temperatures. In addition, the effect of temperature on enzyme stability was studied by incubating purified enzyme for 30 min at temperatures ranging from 30°C to 75°C. After this treatment, residual enzyme activity was determined under standard conditions. For pH dependency, the dehydrogenase activity was assayed in 50 mM sodium acetate buffer (pH 3.5 to 5.0) or 50 mM sodium maleate buffer (pH 6 to 7.0) at 40°C. For pH stability, the purified protein was incubated in the appropriate buffer for 1 h before the assay.

To reach saturation, the kinetics measurements with l-fucose as the substrate were performed at concentrations ranging from 0.1 to 1 M. Experiments were conducted at 40°C in 50 mM sodium acetate buffer at pH 5.5 under the standard reaction conditions with 50 μM cytochrome *c*, 1 mM CaCl_2_, and 4 μM PQQ. The kinetic parameters of recombinant *Tr*AA12 were determined from a nonlinear regression model using the Michaelis-Menten equation. Three replicates were taken for each point.

### Crystallization and data collection.

The first crystals of *Tr*AA12 were obtained by the sitting drop method in a commercial screen (Stura; Molecular Limited Dimension, Suffolk, UK) containing 60% polyethylene glycol (PEG) 550 monomethyl ether (MME), 0.1 M HEPES, pH 8.2. This commercial condition was optimized, and the best crystallization conditions were 55 to 60% PEG 550 MME, 0.1 M HEPES, pH 8.0 to 8.5. The protein concentration for crystallization was 24 mg ml^−1^ in the purification buffer. The PEG 550 MME concentration was sufficient to protect the crystals during nitrogen flash freezing. Iodide crystals were obtained by use of a 1- to 5-min soak in reservoir solution to which 1 M potassium iodide was added, followed by a back soak and then freezing. Several soaks were done using calcium, PQQ, and l-fucose to obtain a holoenzyme structure. The final concentrations in the drop during the soaking were approximately 1 mM CaCl_2_, 5 μM PQQ, and 500 mM l-fucose. Crystals were collected at the French national synchrotron facility, Soleil, on the Proxima 2 beamline for the native data set and on the Proxima 1 beamline for the iodide crystal. The crystals containing calcium were collected at ESRF on the ID30B beamline.

### Structure determination and refinement.

The crystals belong to the P4_3_2_1_2 space group with cell parameters with unit cell dimensions of 83 Å for *a* and *b* and 143 Å for *c*. One protein is present in the asymmetric unit. Single-wavelength anomalous dispersion was used to obtain the phase. Data were collected at a wavelength of 1.653 Å. The initial phases were calculated with the Phaser program suite (46, 47). Phase improvement was done by use of the Parrot tool in the CCP4 program suite (47). Only one iodide ion was found in the structure. The first model was built by use of the buccaneer tool and refined with the Refmac5 tool in the CCP4 program suite (47–49). The Coot program was used to finalize the model building (50). The other structures were solved by molecular replacement using the Refmac5 and Coot programs (47, 49, 50). The assignment of the two calcium ions was verified by using the web server CheckMyMetal (27). The crystallographic data collection and refinement statistics are presented in Table 1.

**TABLE 1:** Crystallographic data collection and refinement statistics

Parameter	Value for^*a*^:		
	*Tr*AA12-KI	*Tr*AA12	*Tr*AA12-Ca^2+^
Data collection statistics			
Beamline	Soleil Proxima 1	Soleil Proxima 2	ESRF ID30B
Wavelength (Å)	1.6531	0.9801	0.9677
Space group	P4_3_2_1_2	P4_3_2_1_2	P4_3_2_1_2
*a*, *b*, *c* unit cell dimensions (Å)	82.78, 82.78, 140.75	82.97, 82.97, 140.71	82.95, 82.95, 146.9
Resolution range (Å)	45.00–1.99 (2.05–1.99)	46.9–2.1 (2.21–2.1)	45.8–1.8 (1.89–1.8)
Completeness (%)	99.5 (93.93)	100 (100)	99.8 (100)
No. of unique reflections	32,304	29,499	45,806
* R*_merge_^*b*^	0.101 (0.705)	0.109 (1.163)	0.051 (0.883)
* R*_pim_^*c*^	0.03 (0.248)	0.042 (0.441)	0.019 (0.335)
Mean〈*I*/σ*I*〉	20.4 (4.2)	11.1 (1.9)	21.5 (2.3)
B-factor from Wilson plot (Å^2^)	33.5	39.5	30.9
Refinement statistics			
* R*_cryst_^*d*^	0.185 (0.271)	0.204 (0.319)	0.195 (0.328)
* R*_free_	0.222 (0.298)	0.256 (0.355)	0.233 (0.399)
No. of free reflections	1,701	1,460	2,383
No. of:			
Protein atoms	2,988	2,987	3,018
Other ligand atoms	129	118	105
Solvent atoms	135	105	136
RMSD from target values			
Bond length (Å)	0.013	0.017	0.013
Bond angle (°)	1.81	1.99	1.75
Chiral vol (Å^3^)	0.137	0.109	0.079
Average B-factor (Å^2^)	35	44.9	38
Ramachandran plot statistics (%)			
Residues in favored regions	95.95	95.71	96.53
Residues in allowed regions	3.29	3.54	2.73
Residues in outlier regions	0.76	0.76	0.74
PDB accession no.	6I1Q	6H7T	6I1T

a The values in parentheses apply to the outermost-resolution shell.

b $R_{\text{merge}}=\sum^{}h\mathrm{kl}\sum^{}i\left[I_{i\left(\mathrm{hkl}\right)}-〈I_{\mathrm{hkl}}〉\right]/\sum^{}h\mathrm{kl}\sum^{}iI_{i\left(\mathrm{hkl}\right)}$, where *I* is an individual reflection measurement and is the mean intensity for symmetry-related reflection.

c $R_{\text{pim}}=\sum^{}h\mathrm{kl}\sqrt{\left(1/n\;-1\right)}\sum^{}i\left|I_{i\left(\mathrm{hkl}\right)}-〈I_{\mathrm{hkl}}〉\right|/\sum^{}h\mathrm{kl}\sum^{}iI_{i\left(\mathrm{hkl}\right)}$.

d $R_{\text{cryst}}=\sum \mathrm{hkl}||F_{\text{o}}|-|F_{\text{c}}||/\sum \mathrm{hkl}|F_{\text{o}}|$, where *F*_o_ and *F*_c_ are the observed and the calculated structure factors, respectively.

### Molecular docking studies.

To estimate the binding mode of the cofactor with the substrate, molecular docking studies were performed using the Autodock Vina program (51). The cofactor (PQQ) and substrate (l-fucose:α-l-fucopyranose) were built and minimized with Gasteiger charges in the UCSF Chimera system (52). Further, minimized cofactor and substrate structures were prepared for docking studies by applying the standard ligand docking protocol. The structure of *Tr*AA12 solved with the active calcium was chosen as the protein model. For protein preparation, all hydrogen atoms added and nonpolar hydrogens were also merged. The Kollman united atom charge and atom type parameters were added. The calcium ion present in the active site was assigned a +2 charge. For the cofactor and substrate, two separate docking sites were set in such way that included the cofactor binding site and the active site. Thirty dock poses were generated for each ligand. The dock conformation with the lowest docking energy, the lowest metal ion distance, and the best superimposition on the reported homologue complex structure (PDB accession number 1CQ1) from the bacterium Acinetobacter calcoaceticus was chosen (12). Further, the interactions of the protein-ligand complex, the hydrogen bonds, and the hydrogen bond length were analyzed in the UCSF Chimera system (52).

### Data availability.

The structures of the iodide adduct for phasing, the native protein, and the catalytic calcium-containing protein may be found in the Protein Data Bank (PDB) under accession numbers 6I1Q, 6H7T, and 6I1T, respectively.
